# An Increased Anticholinergic Drug Burden Index Score Negatively Affect Nutritional Status in Older Patients Without Dementia

**DOI:** 10.3389/fnut.2022.789986

**Published:** 2022-02-08

**Authors:** Esra Ates Bulut, Neziha Erken, Derya Kaya, Fatma Sena Dost, Ahmet Turan Isik

**Affiliations:** ^1^Department of Geriatric Medicine, Adana City Training and Research Hospital, Adana, Turkey; ^2^Unit for Aging Brain and Dementia, Department of Geriatric Medicine, Faculty of Medicine, Dokuz Eylul University, Izmir, Turkey

**Keywords:** anticholinergic activity, cognitive functions, drug burden index, malnutrition, older adults

## Abstract

**Introduction/Aim:**

Anticholinergic drugs, which have severe central and peripheric side effects, are frequently prescribed to older adults. Increased anticholinergic drug burden is associated with poor physical and cognitive functions. On the other side, the impact of anticholinergics on nutritional status is not elaborated in the literature. Therefore, this study was aimed to investigate the effect of the anticholinergic burden on nutrition.

**Materials and Methods:**

Patients who underwent comprehensive geriatric assessment (CGA) 6 months apart were included in the study. Patients diagnosed with dementia were excluded because of the difference in the course of cognition, physical performance and nutrition. Nutritional status and global cognition were evaluated using Mini Nutritional Assessment-short form (MNA-SF), Mini-Mental State Examination (MMSE). Anticholinergic drug burden was assessed with the Drug Burden Index (DBI), enabling a precise dose-related cumulative exposure. Patients were divided into three groups according to DBI score: 0, no DBI exposure; 0–1, low risk; and ≥1, high risk. Regression analysis was performed to show the relationship between the difference in CGA parameters and the change in DBI score at the sixth month.

**Results:**

A total of 423 patients were included in the study. Participants' mean age was 79.40 ± 7.50, and 68.6% were female. The DBI 0 score group has better MMSE and MNA-SF scores and a lower rate of falls, polypharmacy, malnutrition, and risk of malnutrition in the baseline. Having malnutrition or risk of malnutrition is 2.21 times higher for every one-unit increase in DBI score. Additionally, during the 6-month follow-up, increased DBI score was associated with decreased MNA-SF and MMSE score, albumin.

**Conclusions:**

The harmful effects of anticholinergics may be prevented because anticholinergic activity is a potentially reversible factor. Therefore, reducing exposure to drugs with anticholinergic activity has particular importance in geriatric practice.

## Introduction

Older adults are vulnerable to adverse drug reactions and drug-drug interactions because they frequently experience multiple systemic diseases, leading to multiple drug use. Hence, polypharmacy is a significant concern in the management of older patients, and several tools have been developed to review medications, such as Beers Criteria (1), STOPP (Screening Tool of Older Persons' Prescriptions) and START (Screening Tool to Alert to Right Treatment) criteria (2). Basically, anticholinergic effect determines the safety of the drugs. Anticholinergics inhibit acetylcholine action, which has a crucial role in the regulation of several central and peripheral nervous system functions. Significant anticholinergic effects include dry mouth, constipation, tachycardia, urinary retention, drowsiness, and confusion (3).

Mainly, immobilization, urinary incontinence, neurologic and psychiatric comorbidities (dementia, depression, Parkinson's disease, epilepsy) were reported as the most significant risk factors for the anticholinergic prescription (4). This group is also at risk of malnutrition. Due to the effects on gastrointestinal motility and secretions, as well as sedative potency, anticholinergics were reported to be related to gait imbalance, dysphagia, delirium (5, 6). A dry mouth may cause difficulty in swallowing, and decreased gastric motility and constipation may contribute to satiety and anorexia. Drowsiness and confusion may cause dehydration, swallowing problems, and aspiration (5), each of which poses a severe risk for malnutrition.

Moreover, older adults are more susceptible to anticholinergic agents because of physiological and pathological changes with aging, including decreased physiological reserve, pharmacodynamic and pharmacokinetic alterations. The anticholinergic burden is termed the cumulative effect of anticholinergic agents and reported as a predictor of cognitive decline, poorer physical performance, falls, and even mortality (7–11). Currently, the optimal scale has not been determined to qualify total anticholinergic drug burden (12). The Drug Burden Index (DBI) is one of the most commonly used validated risk assessment scales to estimate cumulative exposure to anticholinergic medications (13). Additionally, the DBI enables dose-related measurement of each drug and offers an extensive evaluation of several drug classes. Accumulating evidence indicates DBI is a useful indicator of adverse health outcomes and functionality in older adults (14, 15).

Many studies have investigated the relationship between anticholinergic drug burden and cognitive, physical functions. However, the information about the impact of anticholinergics on the nutritional status is limited. We aimed to show the association between DBI score and malnutrition in older adults.

## Materials and Methods

### Patients and Procedures

The records of patients who applied to the geriatric outpatient clinic between January 2017 and March 2020 were retrospectively reviewed. A total of 1,805 patients' files were screened. Patients who were older than 65 years old and underwent comprehensive geriatric assessment (CGA) (16) two times at 6-month intervals were included in the study. The number of patients who were evaluated twice at the end of 6 months was 811. Exclusion criteria were determined as a severe illness that may impair general health status during the follow-up period, such as acute coronary syndrome, sepsis, acute renal failure, gastrointestinal bleeding, and diagnosis of cancer, immobility, substance and alcohol abuse. Additionally, patients diagnosed with major neurocognitive disorders, including Alzheimer's disease, vascular dementia, Lewy body dementia, and frontotemporal dementia, which cause progressive deterioration of cognitive domains, were excluded from the study because many medications used in the treatment of those patients, including antipsychotics, sedatives, and antidepressants, also increase DBI scores, and the trajectory of dementia differs in functionality, cognition, nutritional status from cognitive intact older adults. As a result, a total of 423 patients, who did not have exclusion criteria and whose records were eligible, were included in the study.

The study was designed as a retrospective observational cohort study. The primary outcome was defined to show the difference of nutritional parameters including Mini-Nutritional Assessment short-form, weight, albumin between patient groups with increased or decreased DBI score at the end of 6 months, and secondary outcomes were global cognitive performance and functionality.

The investigation was conformed to the Declaration of Helsinki and approved by the local ethics committee.

### Comprehensive Geriatric Assessment and Laboratory Measurements

Detailed medication history (including dosage and duration), sociodemographic characteristics, chronic systemic diseases, comprehensive geriatric assessment parameters including Mini-Mental State Examination (MMSE), Geriatric Depression Scale (GDS) (17), Basic (Barthel) and Instrumental (Lawton) Activities of Daily Living (BADL and IADL), Mini-Nutritional Assessment short-form (MNA-SF) were obtained from records of the patients. Falls history in the last year was recorded. Dementia and depression were diagnosed according to the Diagnostic and Statistical Manual of Mental Disorders Fifth Edition (DSM-5) criteria. Comorbidity scores of the patients were calculated using the Charlson Comorbidity Index. Polypharmacy was determined as concurrent five or more drug usage (18). Urinary incontinence was considered positive in having involuntary urinary leakage in the last 3 months except for urinary tract infection (18). Chronic non-cancer pain is accepted as lasting beyond the expected healing time or at least 3–6 months (19).

Laboratory tests including hemogram, albumin, 25-hydroxy vitamin D, vitamin B12, low-density lipoprotein (LDL), high-density lipoprotein (HDL), estimated glomerular filtration rate according to Modification of Diet in Renal Disease (MDRD) Study equation (20) were evaluated.

### Drug Burden Index and Anticholinergic Risk Groups

DBI is calculated for each regularly used drug using a formula calculating the ratio between the prescribed daily dose and the sum of the dose that gives 50% of the maximal effect and the prescribed dose. The total score was achieved by summing the score of each sedative drug separately (21). The calculation was carried out by the web portal software program “Anticholinergic Burden Calculator” (www.anticholinergicscales.es/) (22). The main drug classes that were evaluated for their anticholinergic drug burden were antihistamines, benzodiazepines, antipsychotics, selective serotonin reuptake inhibitors (SSRIs), serotonin and norepinephrine reuptake inhibitors (SNRIs), tricyclic antidepressants (TCAs), alpha blockers, metoclopramide, bladder antimuscarinics and muscle relaxants. Scores of the participants were calculated as a continuous variable. Accordingly, three groups were identified: DBI score 0 (no DBI exposure), DBI score 0–1 (low), and DBI score ≥ 1 (high) (23).

### Nutritional Assessment and Malnutrition

The patients were grouped according to MNA-SF scores: malnourished (<7 points), risk of malnutrition (8–11 points), or well-nourished (≥12 points) (24). Nutritional assessment was conducted twice apart 6-months to the patients such as other CGA parameters.

### Statistical Analysis

**Figure 1:**
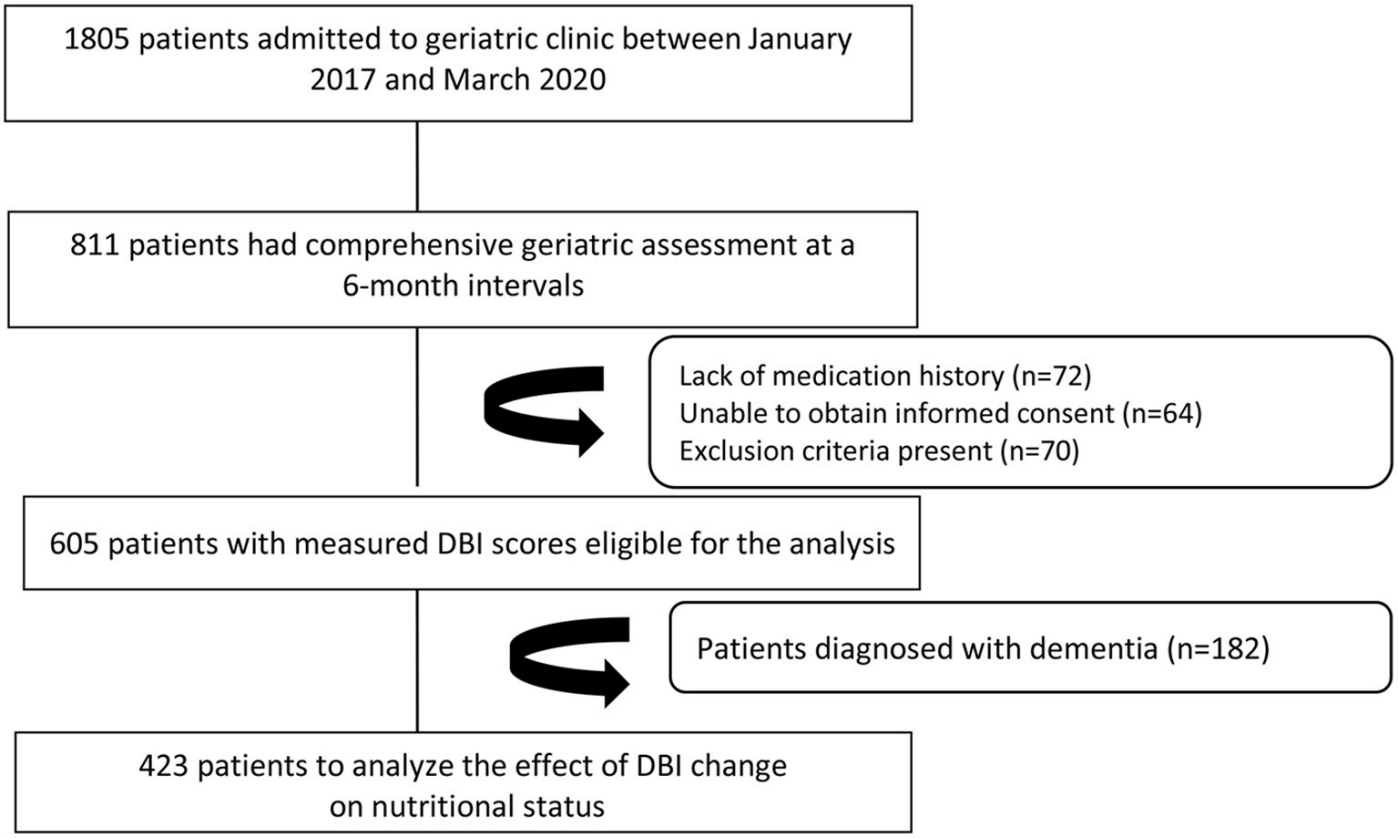
Flowchart of the participants in the study.

Categorical variables were evaluated with chi-square tests. Normal distribution was checked using the Kolmogorov-Smirnov test. Categorical and continuous variables are expressed as percentages (%) and mean ± standard deviations, respectively. The baseline difference of continuous variables between the three groups according to the DBI score was evaluated with the Kruskal-Wallis test and nominal. Categorical variables were evaluated with chi-square test. The relationship between nutritional status and DBI risk groups in the baseline was assessed with logistic regression analysis. In the 6th month patients without dementia were reorganized into three groups: Patients with no DBI exposure from the beginning, patients with decreased DBI score, patients with increased DBI scores. Figure 1 shows a flowchart of the participants in the study. The change in scores was obtained by subtracting the baseline value from the 6th month follow-up value, and the delta (Δ) value was specified for each test score [(Δ) value (6th month evaluation-Baseline evaluation)]. Logistic regression analysis was performed to show the effect of change in DBI score on CGA parameters. Multiple linear regression analysis was conducted to show relationship between change in DBI score and laboratory values. *p*-values lower than 0.05 are accepted as statistically significant. All statistical analyses were performed using the SPSS 25.0 (SPSS Inc.) package program.

## Results

A total of 423 patients were included in the study. Participants' mean age was 79.40 ± 7.50, and 68.6% were female. When the patients were divided into three according to DBI risk score in the baseline, 225 patients had DBI 0 score, 135 had low risk, and 33 had high risk. Age, sex, and educational status were similar between the groups. Patients diagnosed with diabetes mellitus and depression were common in the low and high-risk groups (*p* < 0.05). Compared with patients with a DBI score 0, those in the risk groups had a higher rate of polypharmacy, falls and malnutrition, and higher Charlson Comorbidity Score, whereas lower baseline albumin, and hemoglobin levels. Moreover, the DBI 0 score group has the best values in regards to CGA parameters. Table 1 summarizes the baseline characteristics of the patients.

**Table 1:** Patients' characteristics according to basal DBI risk status.

	**DBI score**			
	**Zero (0)**	**Low risk (0-1)**	**High risk (≥1)**	***p***
	***n***: **225**	***n***: **135**	***n***: **33**	
**Demographics**				
Sex (female%)	68.2	68.9	69.7	0.98
Age (mean ± std deviation)	78.88 ± 7.43	80.51 ± 7.44	78.87 ± 8.03	0.08
Marital status (married) (%)	62.8	60.2	56.3	0.94
Education (years)	8.17 ± 4.93	8.08 ± 4.80	6.38 ± 4.81	0.16
**Comorbidities (%)**				
Depression	22.4	42.2	48.5	**<0.01**
Diabetes mellitus	23.5	28.9	54.5	**<0.01**
Hypertension	65.9	71.1	72.7	0.48
Ischemic cardiac disease	14.5	17.8	21.9	0.46
Charlson comorbidity index	0.82 ± 1.06	1.18+1.38	1.27 ± 1.28	**0.01**
**Geriatric syndromes (%)**				
Polypharmacy	35.3	65.2	87.9	**<0.01**
Urinary Incontinence	38.8	40.0	39.4	0.97
Falls	21.2	34.8	42.4	**<0.01**
Pain	46.6	50.7	59.4	0.34
**Comprehensive geriatric assessment parameters**				
MMSE	27.02 ± 3.28	25.87 ± 4.22	24.50 ± 4.96	**0.01**
GDS	2.52 ± 3.12	3.15 ± 3.02	5.76 ± 4.43	**<0.01**
MNA-SF	12.49 ± 1.96	11.85 ± 2.28	11.70 ± 2.08	**<0.01**
BADL	96.08 ± 6.95	93.70 ± 9.75	88.42 ± 17.20	**<0.01**
IADL	19.99 ± 4.04	18.80 ± 5.00	17.06 ± 6.15	**<0.01**
**Laboratory values**				
Hemoglobin (g/dL)	13.03 ± 1.48	12.73 ± 1.50	12.51 ± 1.52	**0.02**
Albumin (mg/dL)	4.19 ± 0.27	4.07 ± 0.37	4.09 ± 0.41	**0.02**
HDL (mg/dL)	58.63 ± 13.95	55.95 ± 13.65	52.11 ± 14.11	0.06
LDL (mg/dL)	133.85 ± 37.98	133.95 ± 41.44	118.64 ± 34.40	0.27
MDRD (mL/dk)	75.69 ± 18.01	72.67 ± 19.71	73.44 ± 17.65	0.32
Vitamin D (ng/mL)	20.72 ± 14.42	19.25 ± 13.08	22.62 ± 15.60	0.27
Vitamin B12 (pg/mL)	447.01 ± 330.19	537.97 ± 408.66	485.68 ± 375.56	0.25

*MDRD, Modification of Diet in Renal Disease; MMSE, Mini Mental State Examination; GDS, Geriatric Depression Scale (0–15); MNA-SF, Mini Nutritional Assessment (0–12); BADL, Basic Activities of Daily Living (0–100); IADL, Instrumental Activities of Daily Living (0–23). Bold values denote statistical significance at the p < 0.05 level*.

**Figure 2:**
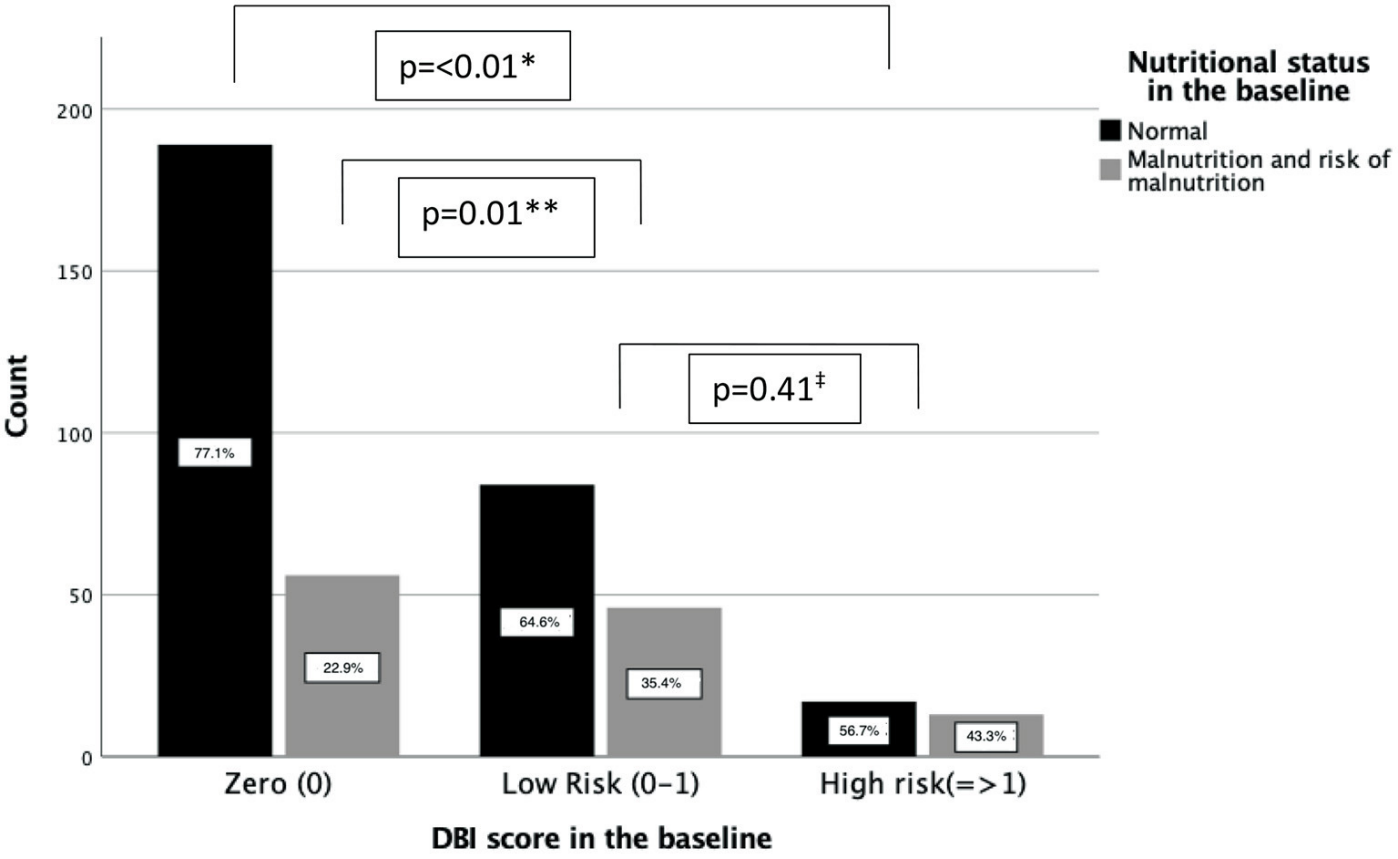
Patients with normal nutritional status within the DBI risk groups. *indicates the *p*-value between DBI high risk and zero groups in terms of malnutrition and risk of malnutrition. **indicates the *p*-value between DBI low risk and zero groups in terms of malnutrition and risk of malnutrition. ^‡^indicates the *p*-value between DBI low risk and high risk groups in terms of malnutrition and risk of malnutrition.

According to the MNA-SF scores, normal nutritional status was statistically higher in the patients with DBI 0 score (*p* < 0.01) compared to both low and high DBI risk groups in the baseline. The total rate of malnutrition and malnutrition risk was similar in the DBI low and high risk groups. Figure 2 shows the nutritional status of the groups in the baseline.

When the relationship between nutritional status and DBI score and number of drugs routinely used was evaluated, it was found that both parameters were associated with increased malnutrition and malnutrition risk. However, the DBI score was found to have a more substantial contribution to the risk. The odds of having malnutrition or risk of malnutrition is 2.21 times greater for every one-unit increase in DBI score. The anticholinergic characteristics of drugs is more substantial impact on the nutritional status instead of total number of drugs used. Table 2 shows the relationship between nutritional status and drug number, and DBI scores.

**Table 2:** Relationship between total number of drugs routinely used, DBI score and nutritional status of patients in the baseline.

	**Malnutrition and malnutrition risk**	
	**OR (95%CI)**	***p***
DBI score	2.21(1.31–3.72)	**0.003**
Drug number	1.13(1.06–1.20)	**<0.001**

*Bold values denote statistical significance at the p < 0.05 level*.

Accordingly, the follow-up data of 414 patients whose DBI score of 0 from the beginning or DBI score changed was analyzed. According to the change in DBI score, three groups were obtained: no DBI exposure from the beginning, decreased DBI scores, and increased DBI scores. Delta scores did not differ between the three groups (Table 3).

**Table 3:** Delta changes of cognition, nutrition and functionality parameters in patients without dementia.

**Δ variables (6th month – baseline score)**	**Zero (0) from beginning (Group1)** ***N***: **100**	**Decreased score (Group2)** ***N***: **125**	**Increased score (Group3)** ***N***: **189**	***P***
**Comprehensive geriatric assessment parameters**				
ΔMMSE	−0.24 ± 2.23	0.47 ± 3.02	−0.18 ± 2.43	0.24
ΔGDS	−0.83 ± 2.82	−0.24 ± 3.31	−0.36 ± 2.22	0.87
ΔBADL	−0.12 ± 3.46	−0.69 ± 6.48	−0.62 ± 6.37	0.77
ΔIADL	−1.01 ± 2.76	−1.08 ± 2.91	−1.33 ± 3.48	0.95
ΔMNA-SF	0.55 ± 2.10	0.51 ± 2.28	0.36 ± 1.52	0.51
**Laboratory values**				
ΔHemoglobin	0.04 ± 0.93	0.12 ± 1.26	−0.05 ± 0.95	0.13
ΔAlbumin	−0.00 ± 0.25	0.04 ± 0.27	−0.08 ± 0.38	0.07
ΔLDL	−0.02 ± 42.43	1.49 ± 47.92	4.96 ± 42.22	0.64
ΔMDRD	0.24 ± 10.37	−1.53 ± 14.36	0.58 ± 15.27	0.79
ΔWeight	−0.10 ± 2.83	−0.91 ± 3.07	−0.36 ± 2.75	0.22

*MDRD, Modification of Diet in Renal Disease; MMSE, Mini Mental State Examination; GDS, Geriatric Depression Scale (0–15); MNA-SF, Mini Nutritional Assessment (0–12); BADL, Basic Activities of Daily Living (0–100); IADL, Instrumental Activities of Daily Living (0–23)*.

When the change in CGA parameters was analyzed, it was shown that every one-unit increase in the DBI score decreases the positive change in nutrition score by 37%. Similarly, the MMSE score decreases by almost 40%. Moreover, a one-unit increase in DBI score also associated with the decrease in albumin level (Table 4).

**Table 4:** The relationship between increase in the DBI score and changes in laboratory and comprehensive geriatric assessment parameters.

	**Beta**	**OR (confidence interval 95%)**	***p***
**Comprehensive geriatric assessment parameters**
MMSE	−0.483	0.617 (0.418–0.911)	**0.015**
GDS	−0.115	0.891 (0.574–1.385)	0.610
MNA-SF	−0.460	0.631 (0.450–0.884)	**0.008**
BADL	−0.347	0.707 (0.466–1.072)	0.102
IADL	−0.104	0.902 (0.585–1.389)	0.639
	**Beta**	**Beta upper**–**lower level**	***p***
**Laboratory values**
MDRD	1.491	(−1.044)–(4.026)	0.248
Hemoglobin	−0.127	(−0.313)–(0.060)	0.183
LDL	4.351	(−4.455)–(13.158)	0.331
Albumin	−0.086	(−0.159)–(−0.014)	**0.020**
Weight (kg)	0.035	(−0.489)–(0.560)	0.895

*MDRD, Modification of Diet in Renal Disease; MMSE, Mini Mental State Examination; GDS, Geriatric Depression Scale; MNA-SF, Mini Nutritional Assessment; BADL, Basic Activities of Daily Living; IADL, Instrumental Activities of Daily Living. Bold values denote statistical significance at the p < 0.05 level*.

## Discussion

The study shows that increased DBI scores are related to geriatric syndromes such as falls, malnutrition, and polypharmacy. Additionally, an increase in the DBI score during the sixth-month follow-up duration is associated with the lower cognitive and nutritional status in cognitively intact older patients. Notably, the DBI score seems more influential on nutritional status than the total drug number.

Parkinson's disease, cognitive impairment, genitourinary conditions, depression, and institutionalization are major predictors of the higher use of anticholinergic drugs. Although older patients are more vulnerable to the side effects of these drugs, anticholinergic drugs are frequently prescribed to older adults. Anticholinergic use prevalence is reported from 22.8 to 55.9% in community-dwelling older adults (12) and more than 79% of inpatients (25). Several tools and guidelines designed to review drugs accept anticholinergic drugs as potentially inappropriate drugs (26). Higher anticholinergic burden is blamed for adverse health outcomes, such as cognitive decline, poorer physical performance, falls, longer length of stay and mortality (11, 12, 27–29). The present study results also support the negative effect of the anticholinergic burden on cognitive functions. The anticholinergic effects on the central nervous system (CNS) areas responsible for movement control, balance, learning, and memory were blamed for the worse outcomes. On the other hand, it is a debate whether increased anticholinergic burden leads to worse outcomes or whether the diseases being treated by the anticholinergic drugs, including neurodegenerative diseases, are responsible for the decline in cognition and physical performance. Therefore, we excluded patients diagnosed with dementia to mitigate the confounding effect of the underlying condition on the course of patients.

Moreover, malnutrition and risk of malnutrition are significant conditions that are reported between 10 and 40% in community dwelling older adults (30). Malnutrition also causes serious health consequences such as falls, osteoporosis, orthostatic hypotension, and mortality (31). Many risk factors, including increased age, dementia, decreased social support, dysphagia, depression, polypharmacy, anorexia were specified to develop inadequate nutrition (32). However, to our knowledge, the relationship between malnutrition and anticholinergic drug burden is not elaborated in literature so far. In this longitudinal study, it was observed that increased anticholinergic load was associated with worsening nutritional status and decreased albumin level which is also an indirect marker for malnutrition (33). The impact of the anticholinergic burden on nutrition may be explained by the gastrointestinal system and cognitive effects of anticholinergic agents (5). One of the well-known peripheral side effects, dry mouth (xerostomia), leads to poor dentition, altered taste, difficulty in deglutition, and digestion (34). Dysphagia makes the development of aspiration more likely and causes serious complications such as aspiration pneumonia. Reduced gastrointestinal motility and secretion contribute to constipation, anorexia, and early satiety. Additionally, central side effects including cognitive impairment, drowsiness, confusion, poor attention, restlessness may lead to swallowing difficulties, dehydration, and aspiration. Sedative effects of the drugs contribute to anorexia (3).

Although the best scale evaluates the cholinergic load is not determined, DBI is a feasible and validated scale (29, 35). Using the DBI offers an assessment of anticholinergic activity with a dose-response model. Thus, the exposure of drugs being administered to a patient could precisely be estimated. Previous studies support that DBI is related to worse cognitive and physical function (6, 36–39). Accordingly, DBI is selected in the present study to evaluate the anticholinergic burden. High and low exposure risk groups were identified to show the impact of cholinergic burden change on nutrition, cognition, and functionality in the longitudinal analysis. Increased DBI scores negatively influence cognitive and nutritional scores.

The study has many strength aspects. First, the anticholinergic burden was evaluated based on medications at the baseline, and changes take into consideration during the 6-month follow-up. Second, all patients were examined in a detailed manner with CGA, including functionality, global cognition, nutritional status, and most of the geriatric syndromes. Third, the study has a large sample size, and a validated scale was used to quantify the anticholinergic burden objectively. Fourth, to the best of our knowledge, the study is the first longitudinal study investigating the relationship between DBI rated anticholinergic burden and nutritional status in older adults. On the contrary, there are limited features of the study. Although patients with major neurocognitive disorders were excluded from the study, we could not adjust all confounding factors such as depression, increasing the risk of exposure to drugs with anticholinergic properties. In addition, the follow-up duration may be shorter to assess long-term effects of anticholinergic drugs. Thus, larger size, long term follow-up studies are needed to clarify the complex relationship between anticholinergic burden and nutritional status in older adults.

## Conclusion

The serious adverse effects may be prevented because the anticholinergic activity is a potentially reversible factor. Therefore, reducing exposure to drugs with anticholinergic activity has particular importance in geriatric practice. Withdrawal of unnecessary drugs (to avoid potential drug interactions) or decrease anticholinergic agents are the main strategies that should be implemented in clinical settings to improve nutritional and cognitive outcomes in older adults. Such tiny interventions provide essential contributions in the management of geriatric cases. Further evidence is warranted to explain the mechanisms that effects of anticholinergic medications on nutrition.

## Data Availability Statement

The datasets used and/or analyzed during the current study are available from the corresponding author on reasonable request.

## Ethics Statement

The studies involving human participants were reviewed and approved by Non-interventional Studies Ethics Committee of Dokuz Eylül University. The patients/participants provided their written informed consent to participate in this study.

## Author Contributions

AI and EA made the study concept and design. NE and FD helped acquisition of data. EA and DK performed analysis, interpretation of data, and draft the manuscript. AI established critical revision of the manuscript for important intellectual content. All authors contributed to the article and approved the submitted version.

## Conflict of Interest

The authors declare that the research was conducted in the absence of any commercial or financial relationships that could be construed as a potential conflict of interest.

## Publisher's Note

All claims expressed in this article are solely those of the authors and do not necessarily represent those of their affiliated organizations, or those of the publisher, the editors and the reviewers. Any product that may be evaluated in this article, or claim that may be made by its manufacturer, is not guaranteed or endorsed by the publisher.
